# Case Report: Case study of 100 consecutive IRONMAN®-distance triathlons—impact of race splits and sleep on the performance of an elite athlete

**DOI:** 10.3389/fspor.2025.1554342

**Published:** 2025-06-26

**Authors:** Beat Knechtle, Luciano Bernardes Leite, Pedro Forte, Marilia Santos Andrade, Ivan Cuk, Pantelis T. Nikolaidis, Volker Scheer, Katja Weiss, Thomas Rosemann

**Affiliations:** ^1^ Medbase St. Gallen Am Vadianplatz, St. Gallen, Switzerland; ^2^ Institute of Primary Care, University Hospital Zurich, Zurich, Switzerland; ^3^ Department of Physical Education, Federal University of Viçosa, Viçosa, Brazil; ^4^ Department of Sports, Instituto Politécnico de Bragança, Bragança, Portugal; ^5^ Department of Sports, Higher Institute of Educational Sciences of the Douro, Penafiel, Portugal; ^6^ CI-ISCE, ISCE Douro, Penafiel, Portugal; ^7^ Research Center for Active Living and Wellbeing (LiveWell), Instituto Politécnico de Bragança, Bragança, Portugal; ^8^ Department of Physiology, Federal University of São Paulo, São Paulo, Brazil; ^9^ Faculty of Sport and Physical Education, University of Belgrade, Belgrade, Serbia; ^10^ School of Health and Caring Sciences, University of West Attica, Athens, Greece; ^11^ Ultra Sports Science Foundation, Pierre-Benite, France

**Keywords:** swimming, cycling, running, sleep, endurance, ultra-Endurance, performance

## Abstract

**Background:**

Long-distance triathletes such as IRONMAN® and ultra-triathletes competing in longer race distances continue to extend ultra-endurance limits. While the performance of 60 IRONMAN®-distance triathlons in 60 days was the longest described to date, we analysed in the present case study the impact of split disciplines and recovery in one athlete completing 100 IRONMAN®-distance triathlons in 100 days. To date, this is the longest self-paced world record attempt for most daily IRONMAN®-distance triathlons.

**Methods:**

To assess the influence of each activity's duration on the total time, the cross-correlation function was calculated for swimming, cycling, running, and sleeping times. The autocorrelation function, which measures the correlation of a time series with itself at different lags, was also employed using NumPy.

**Results:**

The moving average for swimming slightly increased in the middle of the period, stabilizing at ∼1.43 h. Cycling displayed notable fluctuations between ∼5.5 and 7h, with a downward trend toward the end. The moving average for running remains high, between 5.8 and 7.2 h, showing consistency over the 100 days. The moving average for total time hovered at ∼15 h, with peaks at the beginning, and slightly declined in the final days. The cross-correlation between swimming time and total time showed relatively low values. Cycling demonstrated a stronger correlation with total time. Running also exhibited a high correlation with total time. The cross-correlation between sleep time and swimming time presented low values. In cycling, the correlation was stronger. For running, a moderate correlation was observed. The correlation with total time was also high. The autocorrelation for swimming showed high values at short lags with a gradual decrease over time. For cycling, the autocorrelation also began strong, decreasing moderately as lags increased. Running displayed high autocorrelation at short lags, indicating a daily dependency in performance, with a gradual decay over time. The total time autocorrelation was high and remained relatively elevated with increasing lags, showing consistent dependency on cumulative efforts across all activities.

**Conclusions:**

In a triathlete completing 100 IRONMAN®-distance triathlons in 100 days, cycling and running split times have a higher influence on overall times than swimming. Swimming performance is not influenced by sleep quality, whereas cycling performance is. Swimming times slowed faster over days than cycling and running times. Any athlete intending to break this record should focus on cycling and running training in the pre-event preparation.

## Introduction

Over the past decade, the IRONMAN®-distance triathlon covering 3.8 km of swimming, 180 km of cycling and 42.195 km of running has attracted an increasing number of participants globally, encompassing everyone from recreational (age group) triathletes to elite (professional) competitors (1–4). This surge in interest has spurred advancements in scientific research, particularly focused on identifying the key performance-determining factors and understanding the physiological demands imposed by this sport (5). The IRONMAN®'s structure with swimming, cycling, and running in this respective sequence, demands not only elite level physical preparation but also requires comprehensive psychological resilience and effective recovery strategies to sustain performance across all stages of the race (6, 7).

There are differences in performance between age group and professional IRONMAN® triathletes. While the best age group triathletes finish an IRONMAN® triathlon within ∼12–13 h for men and ∼13–14 h for women (8), the fastest professional male IRONMAN® triathletes finish the race below 8 h and the fastest female IRONMAN® triathletes just above 8 h (9). Split times for swimming, cycling and running are 0:50 h:min, 4:10 h:min, and 2:45 h:min for professional male and 0:55 h:min, 4:45 h:min, and 3:10 h:min for professional female IRONMAN® triathletes, respectively (10).

It is observed that completing a single IRONMAN® requires intense physical adaptations (11), but performing several consecutive IRONMAN® distance races over days pushes these limits to an extraordinary level (12, 13). Beyond these physiological responses, additional factors significantly influence athlete performance in long-distance events, including psychological aspects (14), pacing (14–16), the influence of environmental conditions on pacing (17, 18), race strategy (19), sleep quality (20), and recovery strategies (21). External variables, such as weather and environmental factors, can also interact with physiological responses, further affecting daily performance, recovery capacity, and overall health (22–24).

Given the complexity of physiological responses and external factors impacting performance in ultra-endurance events, it is essential to analyze how each discipline (i.e., swimming, cycling, and running) contributes to overall race time (25) and how variables, particularly sleep, affect performance in the context of consecutive events (26, 27). Ultra-endurance studies indicate that sleep is crucial for physical and mental recovery, directly influencing the ability to sustain intense efforts in prolonged competitions (26, 28). However, the interaction between sleep, performance in each discipline, and the cumulative impact over multiple days has yet to be fully investigated.

To date, one of the longest events in multi-day IRONMAN® triathlons has been a case study investigating the completion of 33 IRONMAN®-distance triathlons in 33 days (29). Recently, another triathlete completed even 60 IRONMAN®-distance triathlons in 60 days (12). In the present case study, we investigated the performance dynamics of an IRONMAN® athlete who completed 100 IRONMAN®-distance triathlons in 100 consecutive days. Our analysis focused on understanding (i) the contribution of each split discipline—swimming, cycling, and running—to the total race time and the interrelation among these disciplines, (ii) the influence of sleep duration on daily performance in each division and overall time, and (iii) the performance trends over the 100 days, examining both long-term tendencies and the predictive effect of previous-day performances on subsequent days. The findings would help future athletes intending to complete daily an IRONMAN®-distance triathlon over several consecutive days regarding the optimal pacing strategy.

## Methodology

### Ethical approval

This retrospective study analysing publicly available data was approved by the Institutional Review Board of Kanton St. Gallen, Switzerland, with a waiver of the requirement for informed consent of the participants as the study involved the analysis of publicly available data (EKSG 01/06/2010). The study was conducted in accordance with recognized ethical standards according to the Declaration of Helsinki adopted in 1964 and revised in 2013.

### The athlete

The male triathlete (age 45 years at the time of the event), completed in 2021 during 100 days between March 1st and June 8th daily an IRONMAN®-distance triathlon. He set his own rules as explained in the event website (https://www.ironcowboy.com/conquer-100/). Before this actual record attempt, he had completed 50 IRONMAN®-distance triathlons in 50 days in all 50 US American states (https://www.redbull.com/us-en/iron-cowboy-50-marathons-50-states-50-days). His personal best times are 10:18 h:min for an IRONMAN® triathlon, 4:28 h:min for an IRONMAN®70.3 triathlon, 1:56 h:min for an Olympic distance triathlon, 3:14 h;min for a marathon, and 1:28 h:min for a half-marathon (https://www.ironcowboy.com).

### The event and the data

All locations of the split disciplines were presented on the event website of the athlete (https://www.ironcowboy.com/conquer-100/). The event started on March 1, 2021 and ended on June 9, 2021. Swimming was held in the Lindon Aquatic Center, Lindon, Utah. The athlete was wearing a sleeveless wetsuit. The cycling course of 112.21 miles (180.58 km) was in the region of Provo on the right side of Utah Lake. The run course of 25.12 miles (40.42 km) was held in the region of Lindon, north of the region of Provo. All rules are explained in the event website where drafting was allowed in all three disciplines (https://www.ironcowboy.com/conquer-100/). All split times were measured using a Garmin Forerunner 945 wrist-based GPS watch (https://connect.garmin.com/). Sleep time was measured using a Biostrap EVO PPG sensor (https://biostrap.com/). The device provided daily sleep tracking via photoplethysmographic signals. The athlete's mean sleep duration over the 100 days was ∼6 h and 44 min per night, based on the data collected by the Biostrap EVO sensor. All data were downloaded from the website of the athlete with his permission (https://www.ironcowboy.com/conquer-100/).

### Statistical analysis

A moving average, calculated with NumPy's convolve function, was used to smooth short-term fluctuations and emphasize underlying trends in the time series. This function performs the discrete, linear convolution of two one-dimensional sequences, allowing us to compute the moving average over a sliding window. To evaluate the relationship between the duration of each activity and the total time, the cross-correlation function was applied to swimming, cycling, running, and sleeping durations. This function measures how two time series are correlated at different time lags and was implemented using NumPy's “correlate” function. Cross-correlation was selected because it allows us to investigate whether the time course of a given activity (e.g., swimming) is temporally associated with fluctuations in total performance, even with a potential delay or anticipation. Additionally, we used the autocorrelation function to assess the internal consistency and temporal structure of each activity, identifying potential cycles or repeated patterns in performance. While cross-correlation analyzes the relationship between two distinct variables, autocorrelation evaluates how a variable relates to itself over time. Autocorrelation was chosen as it enables the detection of temporal dependencies within the same variable over time, which is critical in understanding pacing or fatigue patterns across repeated efforts. These analyses allowed us to characterize temporal dependencies and interactions between activities and total performance time across the 100-day period. All analyses were conducted in Python.

## Results

### Moving average of performance in modalities

**Figure 1:**
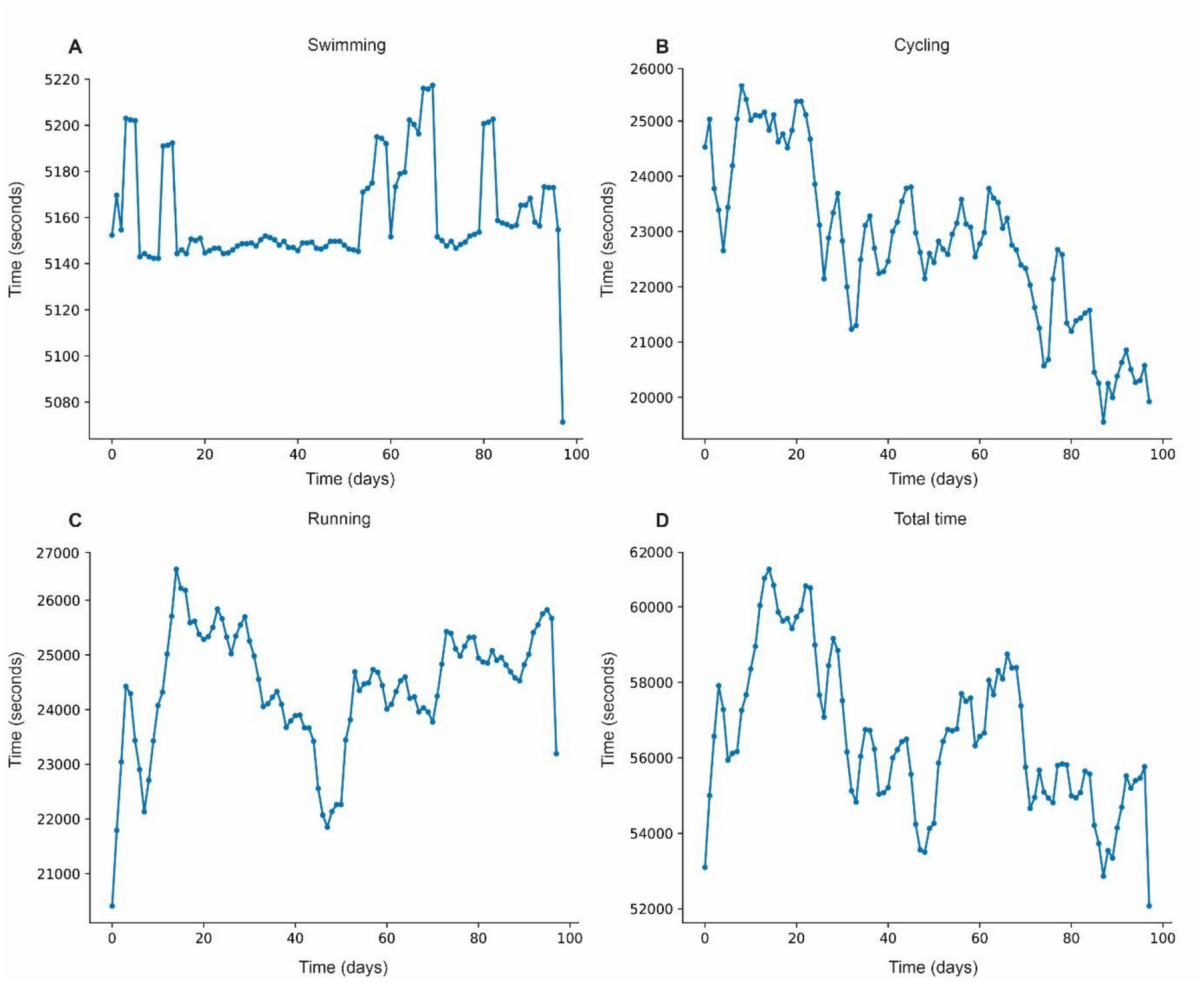
Moving average of performance in modalities. **(A)** Moving average for swimming. **(B)** Moving average for cycling. **(C)** Moving average for running. **(D)** Moving average for total time.

The moving average for swimming shows a slight increase in the middle of the period, stabilizing at ∼5,148 s (Figure 1A). Cycling displays notable fluctuations between ∼19,800 and ∼25,200 s, with a downward trend toward the end (Figure 1B). The moving average for running remains high, between ∼20,880 and ∼25,920 s, showing consistency over the 100 days (Figure 1C). The moving average for total time hovers ∼54,000 s, with peaks at the beginning and a slight decline in the final days (Figure 1D).

### Cross-correlation between total time and modalities

**Figure 2:**
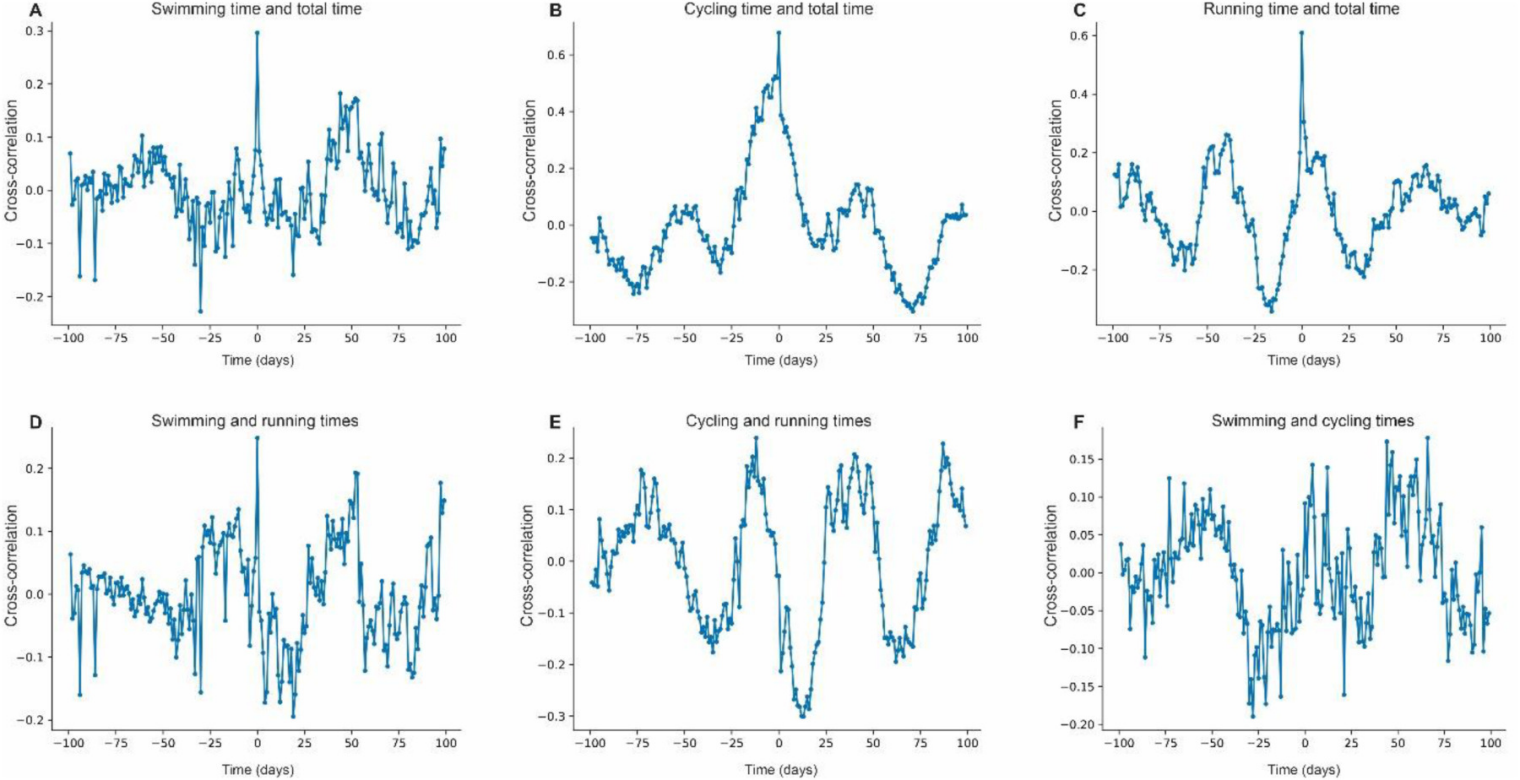
Cross-correlation between total time and modalities. **(A)** Cross-correlation between swimming time and total time. **(B)** Cross-correlation between cycling time and total time. **(C)** Cross-correlation between running time and total time. **(D)** Cross-correlation between swimming and running times. **(E)** Cross-correlation between cycling and running times. **(F)** Cross-correlation between swimming and cycling times.

The cross-correlation between swimming time and total time shows relatively low values, with peaks around 0.2–0.3 (Figure 2A). Cycling demonstrates a stronger correlation with total time, with values between 0.4 and 0.6 (Figure 2B). Running also exhibits a high correlation with total time, reaching up to 0.6 (Figure 2C). The correlation between swimming and running presents low values, around 0.2 (Figure 2D), while the correlation between cycling and running is moderate, reaching 0.3 (Figure 2E). The relationship between swimming and cycling shows moderate peaks of up to 0.2 (Figure 2F). These results indicate a stronger association between cycling and running than with swimming, which appears to be more independent.

### Cross-correlation between sleep and modalities

**Figure 3:**
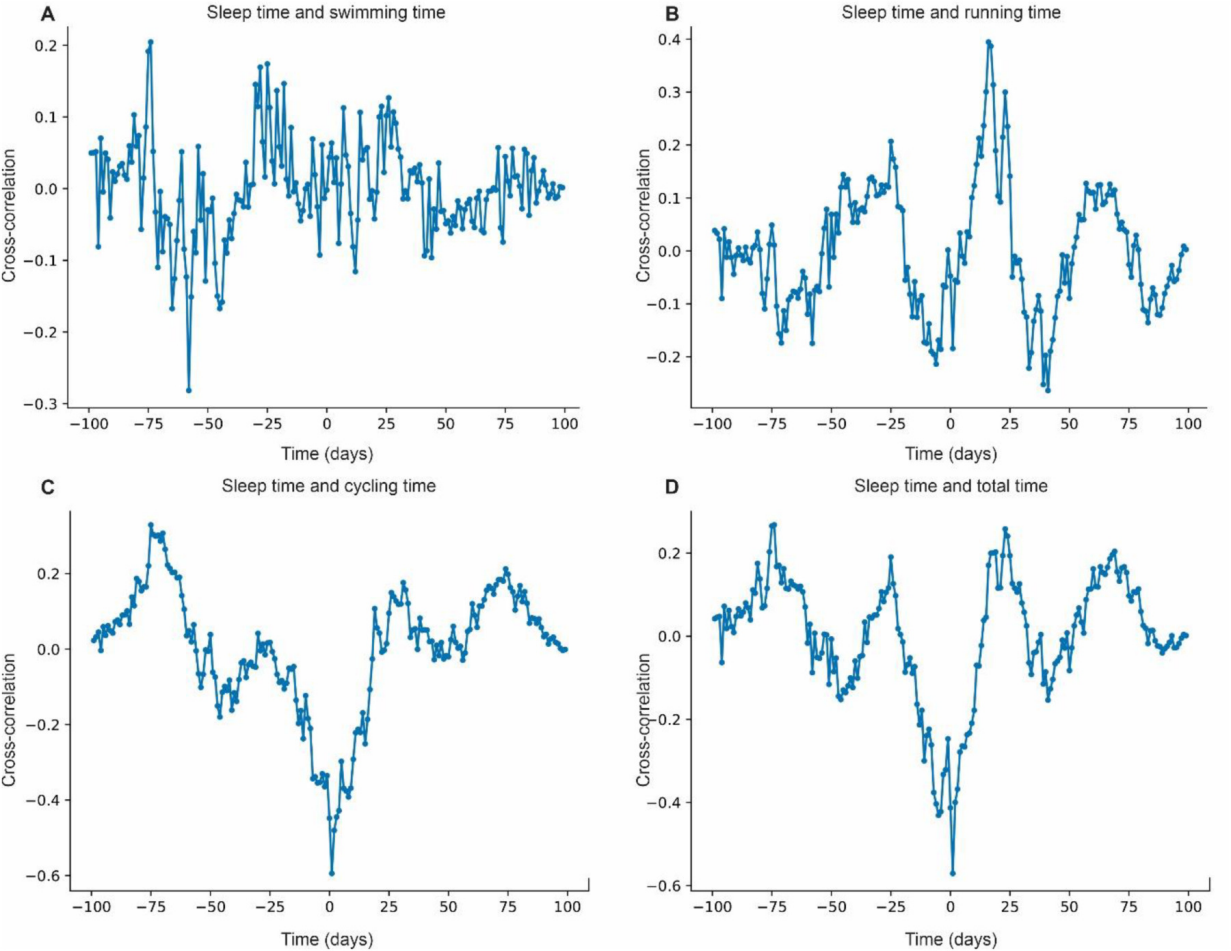
Cross-correlation between sleep time and performance modalities. **(A)** Cross-correlation between sleep time and swimming time. **(B)** Cross-correlation between sleep time and running time. **(C)** Cross-correlation between sleep time and cycling time. **(D)** Cross-correlation between sleep time and total time.

The cross-correlation between sleep time and swimming time presents low values, between 0.2 and −0.2 (Figure 3A). In cycling, the correlation is stronger, ranging from 0.4 to 0.6 (Figure 3B). For running time, a moderate correlation is observed, with peaks between 0.3 and 0.4 (Figure 3C). The correlation with total time is also high, reaching values between 0.4 and 0.6 (Figure 3D). These results indicate a higher correlation of sleep with cycling and total time.

### Autocorrelation analysis of performance in modalities

**Figure 4:**
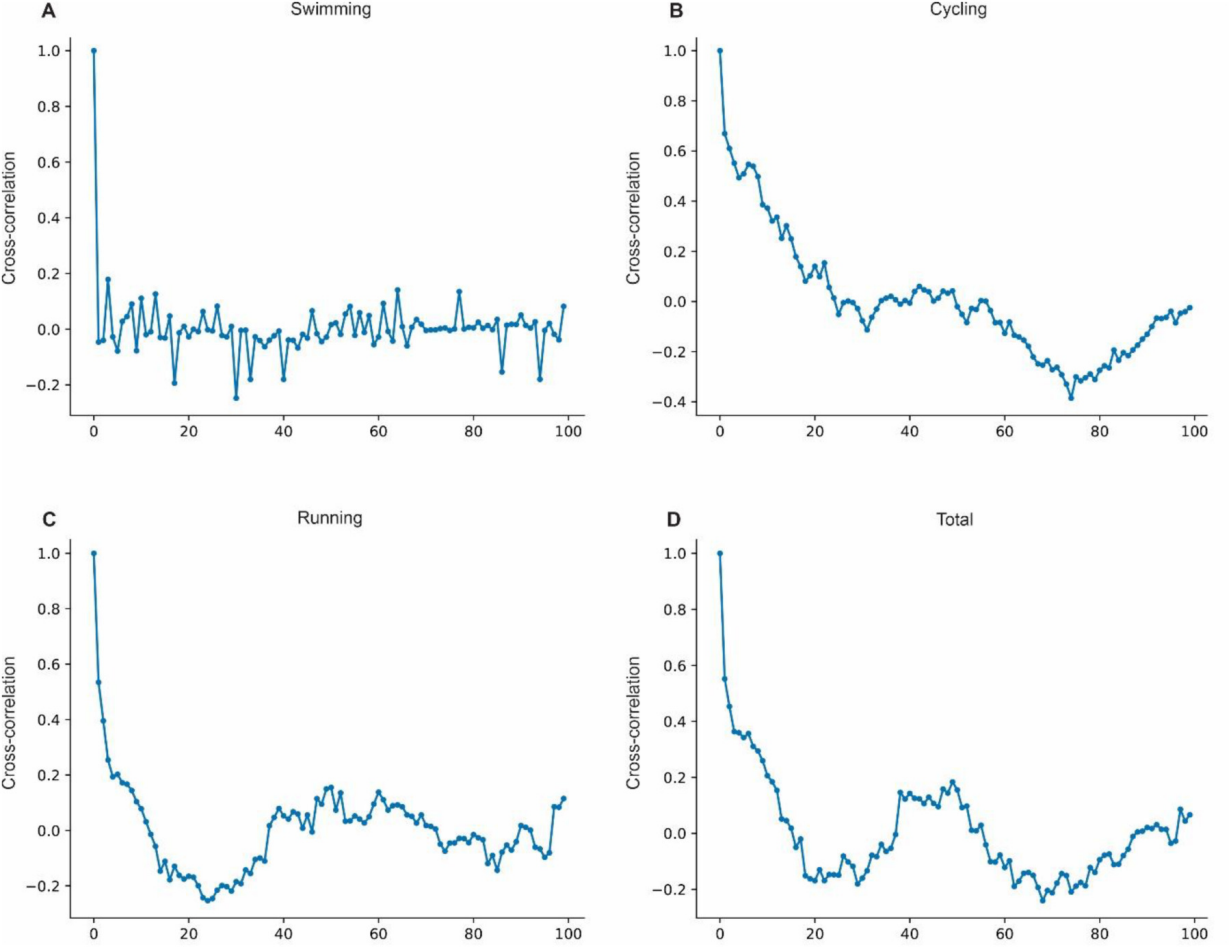
Autocorrelation analysis of performance in modalities. **(A)** Autocorrelation for swimming. **(B)** Autocorrelation for cycling. **(C)** Autocorrelation for running. **(D)** Autocorrelation for total time.

The autocorrelation for swimming shows high values at short lags, close to 1 at lag 0, with a gradual decrease over time (Figure 4A). For cycling, the autocorrelation also begins strong, close to 1 at lag 0, decreasing moderately as lags increase (Figure 4B). Running displays high autocorrelation at short lags, indicating a daily dependency in performance, with a gradual decay over time (Figure 4C). The total time autocorrelation is high at lag 0 and remains relatively elevated with increasing lags, showing consistent dependency on cumulative efforts across all activities (Figure 4D). Notably, swimming exhibits the most rapid decay in autocorrelation, while cycling and total time show a slower decline, and running presents a moderate decay over longer periods.

## Discussion

### Moving average of performance by split disciplines

During the 100 days, swimming performance declined, cycling performance improved, and running performance remained relatively stable. The total time per day increased in the first days and decreased in the last days. The most likely explanation for the increase in cycling performance is the fact that the athlete started drafting after a certain time in the event. In multi-day triathlons, swimming performance tends to deteriorate over extended periods of competition due to the accumulation of fatigue, which negatively impacts stroke efficiency and overall performance. In a case study of a triathlete completing 33 IRONMAN®-distance triathlons in 33 days*,* a similar pattern was observed during multi-day events, where swimming times progressively increased due to the repetitive strain (29). The increase in swimming times in this case could be attributed to a gradual decline in physical and mental energy as the event progressed. Swimming performance might also have changed due to biomechanical changes due to neuromuscular fatigue as well as general fatigue (30, 31).

Cycling performance, on the other hand, improved over time, likely due to the athlete's strategic decision to incorporate drafting after the initial days of the event. Especially, the effect of drafting increases with the position in the group of cyclists (32). Furthermore, drafting has many positive physiological effects (33), where the cyclists use to save about 7% of mechanical power for uphill (33), 4%–42% of drag saving in a velodrome (34), and in single pacelines configurations, the drag reduces about 68% (35). However, for the present study, these conditions were impossible to control.

Drafting, which reduces air resistance and minimizes the energy required to maintain speed, allows for faster cycling splits, as demonstrated by Abbiss et al., who found that drafting significantly lowered energy expenditure and increased performance in long-distance cycling events (17). This effect could explain the observed improvement in cycling times, which showed a notable reduction in total cycling time as the event progressed. Cycling time typically decreased by 5%–10% in the later days, similar to the improvement reported in a case study of a triathlete completing 60 IRONMAN®-distance triathlons in 60 days (12).

Running performance remained stable, with minimal fluctuations throughout the 100 days. This stability could be due to the athlete's adaptive pacing strategy, where the most taxing leg—running—requires careful energy management to prevent early fatigue. As Wu et al. (14) found, runners in long-duration triathlons often adopt a conservative approach, ensuring consistent pacing to avoid dramatic fluctuations in performance, mainly when dealing with cumulative fatigue from the prior swim and cycle legs. Despite fatigue, the athlete maintained relatively stable run times, reflecting a strategic approach to avoid overexertion. The total time per day increased in the early days, likely due to the athlete's adjustment to the repetitive nature of the event, and later decreased as the athlete adapted to the physical strain (12). A similar pattern was reported by Kisiolek et al. (26), where athletes experienced higher cumulative times at the start of ultra-endurance events, followed by a decrease as they optimized their performance and recovery strategies. A reduction in total time of up to 10%–15% by the final days was observed, largely attributed to improved recovery and pacing efficiency.

The athlete's ability to sleep and recover effectively during the event likely contributed to this decrease in total time, with sleep duration correlating positively with performance improvements, as found by Dallam et al. (22). The physiological mechanism underlying this observation was the positive role of sleep on neural, metabolic, and immune-endocrine functions (36). Accordingly, it was previously supported that an increase of sleep duration at night or through napping could ameliorate physical performance in athletes (37). The improvement in cycling and stable running times, despite the high volume of consecutive IRONMAN®-distance races, also supports the findings of Nikolaidis et al. (27), who emphasized the importance of recovery strategies and pacing in maintaining ultra-endurance performance. Therefore, the trends observed in this case align with existing literature on ultra-endurance events, where athletes adapt to the physical and mental stressors of consecutive events, resulting in optimized pacing and recovery strategies that contribute to improved performance over time.

Pacing in a marathon is influenced by different variables such as gender, age, performance, pack, and physiological and psychological factors (38). Pacing during a multi-day event with running a marathon daily showed no major variations between days (39). In multi-day IRONMAN®-distance triathlons, the cycling split had an influence on the subsequent running split (40).

### Cross-correlation between split and total times

Over the 100 days, we found a stronger association between cycling and running than with swimming, which appeared to be more independent. This can be justified by considering the physiological demands and relationships between these disciplines in ultra-endurance events. Cycling and running are more physically demanding and closely related in terms of overall endurance and energy systems activities (41). Cycling and running performance in endurance athletes are often correlated due to the shared muscular endurance required, as both rely heavily on lower body strength and aerobic capacity (42). The stronger association between cycling and running in the present study could be attributed to the fact that both disciplines require a sustained effort and similar muscle groups, resulting in more consistent performance across the two activities (41). In contrast, swimming, being a non-weight-bearing activity, demands different energy systems and muscle groups, particularly engaging the upper body, and tends to be more independent of the other two activities (43). This is consistent with findings in ultra-endurance triathlon studies, where the correlation between swim times and other race splits (such as cycling and running) tends to be lower due to the different physiological demands each discipline places on the body (29, 44, 45). Regarding, swimming, physiological issues will arise but biomechanical and neuromotor factors are also likely to influence the athlete's ability to maintain swimming performance (31, 46). Therefore, while cycling and running exhibit a stronger association due to shared endurance and muscular requirements, swimming's independent nature reflects its distinct physiological demands, leading to a lower correlation with the other race splits (41).

### Cross-correlation between sleep and split disciplines

We found a low correlation for swimming, a strong correlation for cycling, a moderate correlation for running and a high correlation for total times, indicating a higher correlation of sleep with cycling and total time. The high correlation between sleep and total time further supports the idea that sleep recovery plays a critical role in overall performance across all disciplines (47). Studies, such as those by Nikolaidis et al. (27) and Kisiolek et al. (26) have shown that sleep duration directly impacts performance in ultra-endurance events, with sleep correlating strongly with the ability to maintain overall race performance across multiple days (48). Therefore, a higher amount of sleep correlates with better total race times, reflecting the importance of rest in sustaining energy levels for both the athlete and their cumulative performance (49). However, based upon existing knowledge, not only the duration of sleep is important, but also the quality of sleep (50) However, in the present study, the sleep duration was considered due to the participant data collection system.

Cycling and running, which constitute the majority of an IRONMAN® race (∼52% and ∼35% of total race time, respectively (51), involve repetitive high-impact movements that can lead to muscle strain and joint stress (44). In contrast, swimming, which accounts for only about 11% of the race time, is a low-impact activity that generally exerts less stress on the musculoskeletal system (52). This differential impact is further supported by findings that highlight the oxidative stress and muscle damage associated with prolonged cycling and running, which are less pronounced in swimming (53).

Cycling, in particular, involves a continuous and prolonged effort that requires sustained physical and mental engagement for several hours per day (54, 55). These characteristics make it especially sensitive to the effects of reduced sleep. The high correlation between sleep duration and cycling time may reflect the challenges of maintaining energy, coordination, and pacing when sleep is insufficient—factors that are especially critical in endurance cycling. Previous studies with cyclists (56, 57) and triathletes (26) have shown that a better sleep quality is associated with an improved competitive performance.

In endurance athletes, the physical stress from high-intensity activities such as cycling and running can lead to increased production of reactive oxygen species (ROS) and inflammatory markers, which have been linked to sleep quality issues (53). Chronic inflammation can disrupt sleep patterns, leading to difficulties in achieving restorative sleep, which is essential for recovery and performance (58). It has been shown that athletes experiencing higher levels of inflammation report poorer sleep quality, which can further exacerbate fatigue and hinder recovery (59). This cycle of inflammation and sleep disruption can be particularly detrimental for Ironman athletes, who rely on optimal recovery to maintain their training regimens and performance levels (60).

### Autocorrelation analysis of performance in the split disciplines

We found that swimming exhibited the most rapid decay in autocorrelation, while cycling and total time showed a slower decline, and running presented a moderate decay over longer periods. Swimming, as the first discipline in an Ironman event, tends to have a unique performance pattern characterized by a rapid decline in autocorrelation. This can be attributed to the shorter duration of the swim segment, which typically constitutes only about 11% of the total race time (52, 61). As a result, even small absolute fluctuations in swimming performance may represent proportionally large variations relative to the segment's total time, leading to greater statistical variability across days (62). Additionally, the swimming segment is often influenced by factors such as water conditions and the absence of buoyancy aids like wetsuits, which can further exacerbate performance variability (63). This combination of short duration and sensitivity to external factors may explain the rapid decay in autocorrelation observed in swimming.

In contrast, cycling showed a slower decline in autocorrelation. This discipline accounts for the majority of the race time (approximately 52%) and allows for more strategic pacing and energy management (52, 61). The longer duration of cycling provides athletes with the opportunity to stabilize their performance over time, resulting in a more gradual decay in autocorrelation. Studies have indicated that cycling performance has improved over the years, suggesting that athletes are becoming more adept at managing their energy and pacing during this segment (64, 65). Running, while presenting a moderate decay in autocorrelation, reflects a different set of challenges. As the final discipline, running performance can be significantly affected by the cumulative fatigue from the previous segments (66). The variance in running times among athletes is notable, with some studies indicating that running performance can fluctuate more than swimming and cycling due to the physiological demands placed on the body at this stage of the race (67, 68). This moderate decay in autocorrelation suggests that while running performance is stable, it is still susceptible to the effects of fatigue and pacing strategies employed during the earlier segments.

### Strength, weakness, and implications for future research

This case study is not free of limitations. Overall, the athlete decided during the course of the event to ask friends to cycle with him in order to be able to draft. This helped him to achieve faster cycling split times and, consequently, also faster running split times resulting in faster total times. Adopting this practice, his overall daily hours became reduced and he could have more sleep and recovery. A further limitation is that we have no data about the athlete's nutrition or hydration. Furthermore, the athlete set his own rules for his event and—based on the raw data of the split times—after day 20 he was drafting because his cycling split times became considerably faster. This drafting had for sure an effect on the following marathon and on overall time. This detail matters as it could skew the data. Aspects such as temperature (69), altitude (70) and psychological strain (71) could not be considered. Uncontrolled factors like mental fatigue (72), injury risk (73), and nutrition tracking (74) were also not considered. Strength of this case study was its novelty as it provided a unique dataset to study the interplay between sleep, pacing and performance. Future research should examine this topic in a large sample of athletes.

### Practical applications

For athletes and coaches, any athlete intending to complete several daily IRONMAN®-distance triathlons in a row needs to carefully plan the single stages swimming, cycling, and running, in order to have enough recovery time for sleep. Overall, the focus should be on cycling and an even pacing should be obtained. More specifically, any athlete intending to break this record should focus on cycling and running training in the pre-event preparation.

## Conclusion

In summary, in a triathlete completing 100 IRONMAN®-distance triathlons in 100 days, the cross-correlation between split times and total times indicated a stronger association between cycling and running than with swimming, which appeared to be more independent. The cross-correlation between sleep and split times showed a higher correlation of sleep with cycling and total time than with swimming and running. The auto-correlation analysis revealed that swimming exhibited the most rapid decay in autocorrelation, while cycling and total time showed a slower decline, and running presented a moderate decay over longer periods. Future case studies should include nutrition and hydration strategy, environmental conditions, psychological aspects (e.g., mental fatigue, motivation) and overuse injuries which all might have an influence on split and overall performance.

## Data Availability

The raw data supporting the conclusions of this article will be made available by the authors, without undue reservation.

## References

[1] Stiefel M Knechtle B Lepers R. Master triathletes have not reached limits in their ironman triathlon performance. Scand J Med Sci Sports. (2014) 24(1):89–97. 10.1111/j.1600-0838.2012.01473 22582950

[2] Lepers R Rüst CA Stapley PJ Knechtle B. Relative improvements in endurance performance with age: evidence from 25 years of Hawaii ironman racing. Age. (2013) 35(3):953–62. 10.1007/s11357-012-9392-z 22367579 PMC3636391

[3] Lepers R Knechtle B Stapley PJ. Trends in triathlon performance: effects of sex and age. Sports Med. (2013) 43(9):851–63. 10.1007/s40279-013-0067-4 23797729

[4] Knechtle B Thuany M Valero D Villiger E Nikolaidis PT Cuk I Europe has the fastest ironman race courses and the fastest ironman age group triathletes. Sci Rep. (2024) 14(1):20903. 10.1038/s41598-024-71866-6 39245697 PMC11381508

[5] Weiss K Valero D Andrade MS Villiger E Thuany M Knechtle B. Cycling is the most important predictive split discipline in professional ironman® 70.3 triathletes. Front Sports Act Living. (2024) 6:1214929. 10.3389/fspor.2024.1214929 38390230 PMC10881807

[6] Bales J Bales K. Triathlon: how to mentally prepare for the big race. Sports Med Arthrosc Rev. (2012) 20(4):217–9. 10.1097/JSA.0b013e31825efdc5 23147092

[7] Olmedilla A Torres-Luque G García-Mas A Rubio VJ Ducoing E Ortega E. Psychological profiling of triathlon and road cycling athletes. Front Psychol. (2018) 9:825. 10.3389/fpsyg.2018.00825 30022957 PMC6040157

[8] Knechtle B Valero D Villiger E Thuany M Cuk I Forte P Cycling and running are more predictive of overall race finish time than swimming in IRONMAN® age group triathletes. Sports Med Open. (2025) 11(1):30. 10.1186/s40798-025-00835-8 40153133 PMC11953511

[9] Knechtle B Thuany M Valero D Villiger E Nikolaidis PT Andrade MS The association of origin and environmental conditions with performance in professional IRONMAN triathletes. Sci Rep. (2025) 15(1):2700. 10.1038/s41598-025-86033-8 39837943 PMC11751080

[10] Knechtle B Cuk I Villiger E Forte P Thuany M Andrade MS Performance and pacing of professional IRONMAN triathletes: the fastest IRONMAN world championship ever-IRONMAN Hawaii 2022. Sci Rep. (2023) 13(1):15708. 10.1038/s41598-023-42800-z 37735607 PMC10514275

[11] Gulbin JP Gaffney PT. Ultraendurance triathlon participation: typical race preparation of lower level triathletes. J Sports Med Phys Fitness. (1999) 39(1):12–5. 10230162

[12] Knechtle B Cuk I Andrade MS Nikolaidis PT Weiss K Forte P Case report: differences in self-selected pacing in 20, 40, and 60 ironman-distance triathlons: a case study. Front Sports Act Living. (2024) 6:1155844. 10.3389/fspor.2024.1155844 39351144 PMC11439664

[13] Anderson T van Mourik RA Martin KJ Eijsvogels TMH Longoria KA. 100 long-distance triathlons in 100 days: a case study on ultraendurance, biomarkers, and physiological outcomes. Int J Sports Physiol Perform. (2023) 18(4):444–53. 10.1123/ijspp.2022-0327 36898387

[14] Wu SS Peiffer JJ Brisswalter J Nosaka K Abbiss CR. Factors influencing pacing in triathlon. Open Access J Sports Med. (2014) 5:223–34. 10.2147/OAJSM.S44392 25258562 PMC4172046

[15] Hausswirth C Brisswalter J. Strategies for improving performance in long duration events: olympic distance triathlon. Sports Med. (2008) 38(11):881–91. 10.2165/00007256-200838110-00001 18937520

[16] Knechtle B Käch I Rosemann T Nikolaidis PT. The effect of sex, age and performance level on pacing of ironman triathletes. Res Sports Med. (2019) 27(1):99–111. 10.1080/15438627.2018.1546703 30418036

[17] Abbiss CR Quod MJ Martin DT Netto KJ Nosaka K Lee H Dynamic pacing strategies during the cycle phase of an ironman triathlon. Med Sci Sports Exerc. (2006) 38(4):726–34. 10.1249/01.mss.0000210202.33070.55 16679990

[18] Pryor JL Adams WM Huggins RA Belval LN Pryor RR Casa DJ. Pacing strategy of a full ironman overall female winner on a course with major elevation changes. J Strength Cond Res. (2018) 32(11):3080–7. 10.1519/JSC.0000000000002807 30161089

[19] Ofoghi B Zeleznikow J Macmahon C Rehula J Dwyer DB. Performance analysis and prediction in triathlon. J Sports Sci. (2016) 34(7):607–12. 10.1080/02640414.2015.1065341 26177783

[20] de Oliveira V Santos D Sinisgalli R Vancini R Costa G Nikolaidis PT Factors associated with perceived performance drops and musculoskeletal injuries in Brazilian recreational triathletes. Eur Rev Med Pharmacol Sci. (2022) 26(16):5651–9. 10.26355/eurrev_202208_29498 36066136

[21] Mujika I Pereira da Silveira F Nosaka K. Blood markers of recovery from ironman distance races in an elite triathlete. J Sports Med Phys Fitness. (2017) 57(7–8):1057–61. 10.23736/S0022-4707.16.06390-8 27139796

[22] Dallam GM Jonas S Miller TK. Medical considerations in triathlon competition: recommendations for triathlon organisers, competitors and coaches. Sports Med (2005) 35(2):143–61. 10.2165/00007256-200535020-00004 15707378

[23] Hermand E Chabert C Hue O. Ultra-endurance events in tropical environments and countermeasures to optimize performances and health. Int J Hyperthermia. (2019) 36(1):752–60. 10.1080/02656736.2019.1635718 31429600

[24] Turris SA Lund A Bowles RR Camporese M Green T. Patient presentations and medical logistics at full and half ironman distance triathlons. Curr Sports Med Rep. (2017) 16(3):137–43. 10.1249/JSR.0000000000000367 28498220

[25] Sousa CV Aguiar S Olher RR Cunha R Nikolaidis PT Villiger E What is the best discipline to predict overall triathlon performance? An analysis of sprint, Olympic, ironman® 70.3, and ironman® 140.6. Front Physiol. (2021) 12:654552. 10.3389/fphys.2021.654552 34025447 PMC8131838

[26] Kisiolek JN Smith KA Baur DA Willingham BD Morrissey MC Leyh SM Sleep duration correlates with performance in ultra-endurance triathlon. Int J Sports Physiol Perform. (2022) 17(2):226–33. 10.1123/ijspp.2021-0111 34627130

[27] Nikolaidis PT Weiss K Knechtle B Trakada G. Sleep in marathon and ultramarathon runners: a brief narrative review. Front Neurol. (2023) 14:1217788. 10.3389/fneur.2023.1217788 37822525 PMC10563314

[28] Roberts SSH Main LC Condo D Carr A Jardine W Urwin C Sex differences among endurance athletes in the pre-race relationships between sleep, and perceived stress and recovery. J Sports Sci. (2022) 40(14):1542–51. 10.1080/02640414.2022.2091345 35767576

[29] Knechtle B Rüst CA Rosemann T Martin N. 33 ironman triathlons in 33 days-a case study. Springerplus. (2014) 3:269. 10.1186/2193-1801-3-269 24926424 PMC4047275

[30] Aquino M Petrizzo J Otto RM Wygand J. The impact of fatigue on performance and biomechanical variables—a narrative review with prospective methodology. Biomechanics. (2022) 2(4):513–24. 10.3390/biomechanics2040040

[31] López-Belmonte Ó Gay A Ruiz-Navarro JJ Cuenca-Fernández F Cejuela R Arellano R. Open water swimming in elite triathletes: physiological and biomechanical determinants. Int J Sports Med. (2024) 45(8):598–607. 10.1055/a-2289-0873 38648801

[32] Edwards AG Byrnes WC. Aerodynamic characteristics as determinants of the drafting effect in cycling. Med Sci Sports Exerc. (2007) 39(1):170–6. 10.1249/01.mss.0000239400.85955.12 17218899

[33] van den Brandt FAP Khudair M Hettinga FJ Elferink-Gemser MT. Be aware of the benefits of drafting in sports and take your advantage: a meta-analysis. Transl Sports Med. (2023) 2023:3254847. 10.1155/2023/3254847 38654910 PMC11022785

[34] Fitton B Caddy O Symons D. The impact of relative athlete characteristics on the drag reductions caused by drafting when cycling in a velodrome. Proc Inst Mech Eng Part P J Sports Eng Technol. (2017) 232(1):39–49. 10.1177/1754337117692280.

[35] van Druenen T Blocken B. Aerodynamic impact of cycling postures on drafting in single paceline configurations. Comput Fluids. (2023) 257:105863. 10.1016/j.compfluid.2023.105863

[36] Lopes TR Pereira HM Bittencourt LRA Silva BM. How much does sleep deprivation impair endurance performance? A systematic review and meta-analysis. Eur J Sport Sci. (2023) 23(7):1279–92. 10.1080/17461391.2022.2155583 36472094

[37] Cunha LA Costa JA Marques EA Brito J Lastella M Figueiredo P. The impact of sleep interventions on athletic performance: a systematic review. Sports Med Open. (2023) 9(1):58. 10.1186/s40798-023-00599-z 37462808 PMC10354314

[38] Sha J Yi Q Jiang X Wang Z Cao H Jiang S. Pacing strategies in marathons: a systematic review. Heliyon. (2024) 10(17):e36760. 10.1016/j.heliyon.2024.e36760 39281580 PMC11400961

[39] Cuk I Markovic S Weiss K Knechtle B. Running variability in marathon-evaluation of the pacing variables. Medicina (Kaunas). (2024) 60(2):218. 10.3390/medicina60020218 38399506 PMC10890654

[40] Weiss K Sousa CV Thuany M Cuk I Nikolaidis PT Knechtle B. Differences in pacing during cycling and running in ultra-triathlons—the example of ‘Swissultra’. Eur Rev Med Pharmacol Sci. (2022) 26(14):4959–68. 10.26355/eurrev_202207_29281 35916791

[41] Millet GP Vleck VE Bentley DJ. Physiological differences between cycling and running: lessons from triathletes. Sports Med. (2009) 39(3):179–206. 10.2165/00007256-200939030-00002 19290675

[42] Etxebarria N Anson JM Pyne DB Ferguson RA. Cycling attributes that enhance running performance after the cycle section in triathlon. Int J Sports Physiol Perform. (2013) 8(5):502–9. 10.1123/ijspp.8.5.502 23347994

[43] Troup JP. The physiology and biomechanics of competitive swimming. Clin Sports Med. (1999) 18(2):267–85. 10.1016/S0278-5919(05)70143-5 10230563

[44] Lepers R Knechtle B Knechtle P Rosemann T. Analysis of ultra-triathlon performances. Open Access J Sports Med. (2011) 2:131–6. 10.2147/OAJSM.S22956 24198579 PMC3781891

[45] Laursen PB Rhodes EC. Factors affecting performance in an ultraendurance triathlon. Sports Med. (2001) 31(3):195–209. 10.2165/00007256-200131030-00004 11286356

[46] Barbosa TM Bragada JA Reis VM Marinho DA Carvalho C Silva AJ. Energetics and biomechanics as determining factors of swimming performance: updating the state of the art. J Sci Med Sport. (2010) 13(2):262–9. 10.1016/j.jsams.2009.01.003 19409842

[47] Vyazovskiy VV. Sleep, recovery, and metaregulation: explaining the benefits of sleep. Nat Sci Sleep. (2015) 7:171–84. 10.2147/NSS.S54036 26719733 PMC4689288

[48] Roberts SSH Teo WP Aisbett B Warmington SA. Extended sleep maintains endurance performance better than normal or restricted sleep. Med Sci Sports Exerc. (2019) 51(12):2516–23. 10.1249/MSS.0000000000002071 31246714

[49] Irwin MR. Sleep disruption induces activation of inflammation and heightens risk for infectious disease: role of impairments in thermoregulation and elevated ambient temperature. Temperature. (2022) 10(2):198–234. 10.1080/23328940.2022.2109932 PMC10274531 37332305

[50] Kohyama J. Which is more important for health: sleep quantity or sleep quality? Children (Basel). (2021) 8(7):542. 10.3390/children8070542 34202755 PMC8304732

[51] Knechtle B Nikolaidis PT. Running in ironman triathlon. In: Canata GL Jones H Krutsch W Thoreux P Vascellari A, editors. The Running Athlete. Berlin, Heidelberg: Springer (2022). p. 209–14. 10.1007/978-3-662-65064-6_23

[52] Figueiredo P Marques EA Lepers R. Changes in contributions of swimming, cycling, and running performances on overall triathlon performance over a 26-year period. J Strength Cond Res. (2016) 30(9):2406–15. 10.1519/JSC.0000000000001335 26808853

[53] da Luz Scheffer D Aurino Pinho C Müller Hoff ML Acordi da Silva L Benetti M Fonseca Moreira JC Impacto do triatlon ironman sobre os parametros de estresse oxidative. Braz J Kinanthropom Hum Performance. (2012) 14(2):174–82. 10.5007/1980-0037.2012v14n2p174

[54] Javaloyes A Mateo-March M Peña-González I Moya-Ramón M. Assessing sleep quality in elite and junior cyclists. Front Sports Act Living. (2024) 6:1369435. 10.3389/fspor.2024.1369435 38752212 PMC11095108

[55] Lahart IM Lane AM Hulton A Williams K Godfrey R Pedlar C Challenges in maintaining emotion regulation in a sleep and energy deprived state induced by the 4800 Km ultra-endurance bicycle race; the Race Across AMerica (RAAM). J Sports Sci Med. (2013) 12(3):481–8. 24149155 PMC3772592

[56] Sargent C Halson SL Martin DT Roach GD. Consecutive days of racing does not affect sleep in professional road cyclists. Int J Sports Physiol Perform. (2022) 17(3):495–8. 10.1123/ijspp.2021-0102 35026733

[57] Roberts SSH Teo WP Aisbett B Warmington SA. Effects of total sleep deprivation on endurance cycling performance and heart rate indices used for monitoring athlete readiness. J Sports Sci. (2019) 37(23):2691–701. 10.1080/02640414.2019.1661561 31526108

[58] Irwin MR Olmstead R Carroll JE. Sleep disturbance, sleep duration, and inflammation: a systematic review and meta-analysis of cohort studies and experimental sleep deprivation. Biol Psychiatry. (2016) 80(1):40–52. 10.1016/j.biopsych.2015.05.014 26140821 PMC4666828

[59] Charest J Grandner MA. Sleep and athletic performance: impacts on physical performance, mental performance, injury risk and recovery, and mental health. Sleep Med Clin. (2020) 15(1):41–57. 10.1016/j.jsmc.2019.11.005 32005349 PMC9960533

[60] Mullington JM Simpson NS Meier-Ewert HK Haack M. Sleep loss and inflammation. Best Pract Res Clin Endocrinol Metab. (2010) 24(5):775–84. 10.1016/j.beem.2010.08.014 21112025 PMC3548567

[61] Barbosa LP Sousa CV Sales MM Olher RDR Aguiar SS Santos PA Celebrating 40 years of ironman: how the champions perform. Int J Environ Res Public Health. (2019) 16(6):1019. 10.3390/ijerph16061019 30897812 PMC6466240

[62] Versey NG Halson SL Dawson BT. Water immersion recovery for athletes: effect on exercise performance and practical recommendations. Sports Med. (2013) 43(11):1101–30. 10.1007/s40279-013-0063-8 23743793

[63] Pla R Raineteau Y Barbier X Aubry A. Physiological key determinants of elite open-water swimmers. Physiologia. (2024) 4(3):305–16. 10.3390/physiologia4030018

[64] Nikolaidis PT Valero D Weiss K Villiger E Thuany M Sousa CV Predicting overall performance in ironman 70.3 age group triathletes through split disciplines. Sci Rep. (2023) 13(1):11492. 10.1038/s41598-023-38181-y 37460563 PMC10352283

[65] Sousa CV Barbosa LP Sales MM Santos PA Tiozzo E Simões HG Cycling as the best sub-8 h performance predictor in full distance triathlon. Sports. (2019) 7(1):24. 10.3390/sports7010024 30669265 PMC6359305

[66] Bentley DJ Cox GR Green D Laursen PB. Maximising performance in triathlon: applied physiological and nutritional aspects of elite and non-elite competitions. J Sci Med Sport. (2008) 11(4):407–16. 10.1016/j.jsams.2007.07.010 17869183

[67] Rico Bini R Canal Jacques T Hunter J Figueiredo P. Biomechanical and physiological implications to running after cycling and strategies to improve cycling to running transition: a systematic review. J Sci Med Sport. (2022) 25(10):861–6. 10.1016/j.jsams.2022.07.006 35871903

[68] Kandel M Baeyens JP Clarys P. Somatotype, training and performance in ironman athletes. Eur J Sport Sci. (2014) 14(4):301–8. 10.1080/17461391.2013.813971 23834510

[69] Weiss K Valero D Villiger E Scheer V Thuany M Aidar FJ Associations between environmental factors and running performance: an observational study of the Berlin Marathon. PLoS One. (2024) 19(10):e0312097. 10.1371/journal.pone.0312097 39413062 PMC11482731

[70] Jeker D Falbriard M Vernillo G Meyer F Savoldelli A Degache F Changes in spatio-temporal gait parameters and vertical speed during an extreme mountain ultra-marathon. Eur J Sport Sci. (2020) 20(10):1339–45. 10.1080/17461391.2020.1712480 31914356

[71] Costello SE Rossiter JRW Howatson G Bell PG O'Neill BV van Someren K Effect of intensified training on cognitive function, psychological state & performance in trained cyclists. Eur J Sport Sci. (2023) 23(7):1334–44. 10.1080/17461391.2022.2097130 35771645

[72] Schiphof-Godart L Roelands B Hettinga FJ. Drive in sports: how mental fatigue affects endurance performance. Front Psychol. (2018) 9:1383. 10.3389/fpsyg.2018.01383 30174627 PMC6107844

[73] Scheer V Krabak BJ. Musculoskeletal injuries in ultra-endurance running: a scoping review. Front Physiol. (2021) 12:664071. 10.3389/fphys.2021.664071 33868030 PMC8044296

[74] Costa RJS Knechtle B Tarnopolsky M Hoffman MD. Nutrition for ultramarathon running: trail, track, and road. Int J Sport Nutr Exerc Metab. (2019) 29(2):130–40. 10.1123/ijsnem.2018-0255 30943823

